# 2489 Perceptions of “translation” and the application of research across disciplines at the University of Michigan

**DOI:** 10.1017/cts.2018.296

**Published:** 2018-11-21

**Authors:** Misty Gravelin, Meagan Ramsey, Kanchan Lota, George Mashour

**Affiliations:** University of Michigan School of Medicine

## Abstract

OBJECTIVES/SPECIFIC AIMS: There is no consensus on what constitutes translational research. To effectively support translation of research into practical settings, universities must determine who is involved, in which disciplines, and what results. In addition, it is unclear whether these researchers would see “translational research” as describing their work. METHODS/STUDY POPULATION: A survey assessing perceptions, successes, and barriers to the application of research was distributed to faculty, fellows, and graduate students within the University of Michigan. This survey included a question on the definition of translational research. RESULTS/ANTICIPATED RESULTS: Investigators of every rank and school participated (n=865), and all schools reported forms of applied research. Over 70% of participants said it was important to use research results beyond academia, and those responses represented diverse successes ranging from product development to artistic endeavors. Common barriers to such as lack of time and funding were also widely experienced. The definitions of translational research were divided between strictly health-oriented or broadly focused application. However, both definitions and familiarity with the term differed by field. DISCUSSION/SIGNIFICANCE OF IMPACT: Translation of research is widespread throughout the university, and many would define translational research to include their research discipline. Strategic university policies could benefit society by enhancing translation and application across many disciplines.

